# Infant mortality inequities for Māori in New Zealand: a tale of three policies

**DOI:** 10.1186/s12939-020-01340-y

**Published:** 2021-01-06

**Authors:** Christopher Rutter, Simon Walker

**Affiliations:** 1 Kenyon College, Gambier, 43022 Ohio USA; 2 University of North Carolina Gillings School of Global Public Health, Chapel Hill, 27599 North Carolina USA; 3 University of Otago, 362 Leith Street, North Dunedin, Dunedin, 9016 New Zealand

**Keywords:** Māori, New Zealand, Race, Inequity, Equity, Health/healthcare, Infant mortality, Policy, Differential risk

## Abstract

**Background:**

The history of infant mortality inequities among Māori in New Zealand provides a remarkable case study for understanding the shortcomings of policy which fails to consider the differential risks associated with disadvantaged groups. Specifically, the failure of the initial 1991 reform in addressing Māori infant health, followed by the relative success of post-1994 policy, demonstrate that disadvantaged populations carry differential social risks which require adjusting policy accordingly. Literature on these policies show that differential risks may include disparities in representation, access to resources, socioeconomic status, and racism. The consideration of differential risks is important in analyzing the underlying causes of inequities and social policy deficiencies.

**Aim:**

To describe and illustrate the need for policy addressing inequities to consider the differential risks associated with disadvantaged groups through an analysis of New Zealand’s Māori infant mortality policy progression.

**Methods:**

The article is a commentary on a series of policies aimed at reducing infant mortality in New Zealand. It analyses three policies and how their differences are linked to the corresponding trends in equity between Māori and non-Māori populations.

**Findings:**

The progression of Māori infant mortality policy clearly demonstrates that equitable social policy must be culturally sensitive and inclusive towards disadvantaged groups, as well as willing to adapt to changing circumstances and shortcomings of current policy. Prior to 1994, health policy which did not account for the differential risks of Māori populations caused inequities in infant mortality to increase, despite infant mortality decreasing on a national level. After policy was adjusted to account for Māori-specific risks in 1994, infant mortality inequities significantly declined. A comprehensive analysis of these policies shows that the consideration of differential risks is highly related to a decrease in corresponding inequities.

**Conclusions:**

As New Zealand, and other countries facing inequities such as the United States and Australia, move forward in constructing policy, they would do well to consider the lessons of how New Zealand policy changed the frequency of infant mortality in Māori populations. The study shows that the consideration of differential risks associated with disadvantaged groups is necessary for policy to successfully address inequities.

## Introduction

The New Zealand healthcare system is best classified as a variation on the Beveridge model, and tends to exhibit outcomes comparable with other developed democracies [1]. However, despite its universal coverage, the New Zealand system is consistently tainted by lower health outcomes in the indigenous Māori and Pacific Islander populations [2]. The reasons for these inequities are multifactorial and interconnected, ranging from racism to socioeconomic disparities to policy [3–6].^1^ In response to this problem, New Zealand implemented a series of reforms designed to equalize outcomes through social policy targeting Māori populations, beginning in the early 1990s [7]. However, the process of reducing inequities has not been a universally successful story. This is particularly evident in the development of policy addressing infant mortality disparities between non-Māori and Māori populations. New Zealand initially established measures designed to address a nationwide problem of high infant mortality, and subsequently adjusted this policy to directly target Māori inequity. As a result, outcomes became considerably more equitable. As such, the sequence of infant mortality policies in New Zealand provides a remarkable case study for understanding important components of successful inequity-reduction. Specifically, the recent reforms highlight the importance of considering the different strains of risk associated with disadvantaged populations. These differential risks include variations in culture, susceptibility to outreach, extraneous factors which indirectly contribute to outcomes such as socioeconomic status and racism, the underlying causes of health problems, and the degree to which risks affect communities. New Zealand targeted these differential risks through equity-focused policy, representative outreach team leaders, targeted safety messages, and culturally sensitive recommendations. By disproportionately focusing on the more disadvantaged Māori population, these policies reflected a ‘proportionate universalism’ approach to equity, where services were allocated at a scale and intensity proportionate to the degree of need [8].

In making significant efforts to anticipate and address these differential social risks, inequity-reduction policy is better able to successfully target disadvantaged communities like the Māori, subsequently decreasing inequities in health outcomes. These efforts tend to generate greater inclusion of disadvantaged groups in making and delivering policy, as well as changing public health strategies to fit the unique circumstances of these groups. While the disparities of New Zealand Healthcare have not been eliminated, there are still valuable lessons to be taken from the country’s efforts to correct infant mortality inequities between Māori and non-Māori populations.

### Defining the problem

In virtually every measurable health outcome, Māori and Pacific Islander populations score significantly lower than their non-Māori non-Pacific counterparts [9, 10]. Commonly, macro-level statistics are used to demonstrate this inequity (Table 1).

**Table 1:** Differences between Māori and non-Māori populations in major public health and demographic criteria

Health and demographic criteria	Māori	Non-Māori	Difference
Life expectancy (years), 2012–2014	73.0 (male) 77.1 (female)	80.3 (male) 83.9 (female)	7.3 (male) 6.8 (female)
Amenable mortality^2^ (rate ratios Māori: Non-Māori), 2015	~ 2 (male) 2.39 (female)	~ 1 (male) 1.00 (female)	2: 1 2.39: 1
Infant mortality (deaths per 1000 live births per year), 2012–2014	5.8	4.6	1.2
Total fertility rate (live births per 1000 women of reproductive age per year), 2014	2.34	1.92 (total NZ population)	0.42
Percent aged < 15 years, 2013	33.7%	18.0%	15.7%
Percent school completion (level 2 +, 15+ years), 2013	45.1%	64.3%	19.2%
Percent unemployed (15+ years), 2013	10.4%	4.0%	6.4%
Percent total personal income < $10,000 (15+ years), 2013	24.1%	18.4%	5.7%
Percent receiving income support (15+ years), 2013	30.4%	13.8%	16.6%
Percent household crowding, 2013	18.6%	7.7%	10.9%

Values derived from the New Zealand Ministry of Health and Stats NZ, which had the most recent publicly available data as of 2019 [9–11]^3^

These major measures of country health are testament to the health inequity problem New Zealand currently has. Over the past three decades, there has been a major push from the New Zealand government to openly acknowledge these inequities and address them through governmental strategies, plans, and policy. Notably there have been three major reforms since the 1990s which together have acknowledged the inequities publicly, committed to collecting data in this area, reallocated funding to favor more disadvantaged areas containing higher Māori populations, and provided public commitments and goals for eliminating these inequities through carefully-crafted policy [12, 13].^4^^,^^5^ However, despite these substantial efforts, benefits have only improved Māori health inequities in certain sectors of healthcare, while notably failing in others. Additionally, even amongst the improved sectors, health outcomes are rarely equable [10].

### Infant mortality: a tale of three policies

Infant mortality provides one example where social policy shifts have somewhat improved health disparities, as Māori outcomes have improved at a faster rate than their non-Māori counterparts since 1996 (Table 2). Importantly, within the larger infant mortality criteria there are two major subcategories of interest: Sudden Unexpected Death in Infancy (SUDI) and Sudden Infant Death Syndrome (SIDS) Mortality. SIDS is more commonly known as ‘cot death’, while SUDI is comprised of SIDS plus fatal sleep accidents. Our analysis of infant mortality primarily looks at these two measures. The risk factors of SIDS and SUDI, which include smoking, bedsharing, and not breastfeeding, are largely social and occur in the home. Because the policies we look at mostly address these risk factors, a majority of the infant mortality data and trends are due to changes in SIDS and SUDI, as opposed to hospital-related infant mortality.

**Table 2:** Infant mortality, SUDI, and SIDS rates in 1996–1998 and 2012–2014 for Māori and non-Māori populations

Infant mortality criteria (deaths per 1000 live births)	1996–1998	2012–2014	Difference (% decrease)
Infant mortality			
Māori	10.2	5.8	−4.4 (43%)
Non-Māori	4.6	4.6	0 (0%)
Sudden unexpected death in infancy (SUDI)			
Māori	3.9	1.3	−2.6 (67%)
Non-Māori	0.8	0.4	−0.4 (50%)
Sudden infant death syndrome (SIDS) mortality			
Māori	3.3	0.5 (nssd)	−2.8 (85%)
Non-Māori	0.7	0.2 (nssd)	−0.5 (71%)

No statistically significant difference between Māori and non-Māori is indicated by the label “(nssd)”. Values are derived from the New Zealand Ministry of Health’s Wai Health Trends Report 2019, which has the most recent publicly available data as of 2019 [10]

Before analyzing this data, it is important to understand the unique nature of the issue, as well as the historical context. Unlike most social policy analyses in healthcare, this issue is largely separated from the health institutions of New Zealand. This is because the risk factors of infant mortality, such as bedsharing and smoking, are primarily related to the home. Hence, access to New Zealand’s largely public health service has less of a role in the inequitable rates of infant mortality between Māori and non-Māori populations. This means that policy reforms need to focus less on treatment funding and more on outreach. In other words, infant mortality reform can be thought of as both educational and social policy in addition to classical health policy, which is more focused on institutions and access.

### The first reform: the Ministry of Health National SIDS prevention campaign of 1991

New Zealand’s infant mortality problem can be understood by looking at the historical progression of three major social policies implemented since the 1980s. During this initial time period, it became apparent that infant mortality rates were drastically higher for New Zealand than other comparable Western democracies [7]. This eventually prompted the Ministry of Health National SIDS Prevention Campaign of 1991, a prevention program which sought to address the major risk factors of SIDS, a subsector of infant.^6^ These risk factors included face-down/prone sleeping, bedsharing (especially with smoking parents), and not breastfeeding. However, while this first reform movement saw non-Māori SIDS rates decrease substantially, the Māori SIDS rates were virtually unchanged. In this respect, the inequities were widened [7].^7^

The primary reason this first reform failed to redress infant mortality inequities was that the educational outreach targeting the three primary risk factors were heavily biased to be more favorably received by non-Māori audiences. There are several aspects to this. Firstly, Māori were not adequately represented nor involved in the prevention campaign. Consequently, messages were less likely to be listened to by the Māori. While there was an increase in Māori turning their children on their back to sleep [14], which is likely the primary cause for the small decline in SIDS in Māori populations, Māori rates of smoking and not breastfeeding, the other main risk factors for SIDS, did not decline to the same degree [7]. While this result can be interpreted in the context of differences in socioeconomic status, it is also possible that these risk factors remained as a product of Māori receiving the information in culturally insensitive ways. This second interpretation is consistent with research that shows a positive racial bias towards one’s own race when internalizing messages. For example, in Davis and High’s research on racial gaps in 2019, it was found that certain groups perceive higher support when acquiring it from members of their own race [15].

Secondly, the messages being put out by the program were culturally and contextually insensitive. For example, the risks associated with infant-parent bedsharing and unsafe blanket use were targeted through anti-bedsharing and anti-blanket messages, both of which are highly valued in Māori culture, and consequently were not well received by Māori groups [16]. This resentment was furthered as a result of messages being delivered primarily by non-Māori workers.

Thirdly, the antismoking campaign was largely unsuccessful. While the absolute decline in smoking was similar for both Māori and non-Māori, the higher smoking prevalence in Māori meant that inequities actually increased [17]. It is likely the combination of nicotine addictions being incredibly hard to overcome, the lack of Māori involvement, and the overrepresented lower socioeconomic factors associated with higher smoking rates (due to higher stress and lower quality of life) all contributed to this increasing inequity.^8^

The failure of New Zealand’s first big infant mortality policy to address inequities demonstrated the shortcomings of social policy that does not take into account the distinct features associated with disadvantaged groups. In this case, there was a lack of Māori inclusion and understanding, in addition to a lack of effort put into addressing underlying problems, such as higher Māori smoking addiction, which is in part a product of socioeconomic factors. Rather, the program sought to address the problem in a uniform way, without recognizing that causes varied by population group. Consequently, while the outcomes improved for the country as a whole, the disparity between the Māori and non-Māori widened.

### The second reform: the Māori SIDS prevention programme

This inequity prompted the creation of the second big reform, the Māori SIDS Prevention Programme, in 1994 [7]. This program intentionally set out to address the deficiencies of the prior reform by appointing Māori program leaders who could connect with Māori on a community level in a highly sensitive manner. It then expanded on the basic social policy and outreach of the early 1990s by creating teams which traveled to these communities to provide support. This new approach made it possible to address the risk factors that the prior policy had not been able to reach. This included a goal of creating smoke-free zones for infants, which required a culturally sensitive push against a highly valued Māori custom of bedsharing [18, 19].^9^

On the whole, the more culturally sensitive approach is thought to have worked. Importantly, it is difficult to make this claim definitively due to the definition of “Māori” expanding soon after the policy’s implementation to include any degree of Māori ancestry, as opposed to the previous criteria of over 50% Māori [7]. This confounds the observed rates by disproportionately increasing the denominator, leading to lower calculated rates. However, it was still concluded that the policy had done well in addressing infant mortality among Māori, as the rates either dropped or remained stagnant [7]. Additionally, it is thought that stagnation is actually a mark of success because the other socioeconomic risk factors for SIDS increased during this time period [7]. Data past the immediate assessments of the policy shows that infant mortality, and two subsections of infant mortality (SIDS and SUDI), decreased at a higher rate among Māori from 1996 to 2014 (Table 2). In fact, this change was so dramatic that by 2012–2014, the specific portion of infant mortality this policy targeted (SIDS) no longer had a statistically significant difference between Māori and non-Māori (Table 2).

While there are some uncertainties around which aspects of the policy were successful, the new approach was overall far more effective than the previous one in addressing inequities. One area in which it is difficult to tell where the benefits came from is smoking. Following the policy, Māori and non-Māori smoking decreased at similar rates, which suggests that smoking was not a factor behind the aligning of infant mortality rates. However, while overall rates had similar trends, young Māori smoking rates actually decreased at a faster rate than non-Māori [10]. Thus, it is difficult to tell if the benefits which we saw with infant mortality and SIDS/SUDI came from other areas of this policy. Additionally, it is possible that even though the overall smoking rates did not decrease for Māori at a faster rate, the creation of smoke-free zones for children might have, which could have contributed to the overall decrease in infant mortality inequities.

This analysis of the second major policy addressing infant mortality is important in that it represents the first major transition from general outcome targets to equity-focused outcome targets, where more inclusive, contextually appropriate, and culturally sensitive methods were able to erase some of the inequities between Māori and non-Māori outcomes. By comparing this policy with the first, one can see the importance of accounting for the different risks factors associated with disadvantaged groups when constructing policy that seeks to close inequity gaps. Analogously, the first policy was like treating two populations with the same general disease using the same antibiotic medicine, despite one of the populations carrying strain resistant to this antibiotic. The comparison between the two policies demonstrates how different populations require different solutions.

### The third reform: Wānanga Wahakura

The third, and final, social policy implemented by New Zealand came as a subset of the second policy, and was coined the Wānanga Wahakura [20]. This policy targeted the SIDS/SUDI risk factors of bedsharing between infants and smoking mothers, which was disproportionately high among Māori [18, 19]. Data showed that bedsharing had not been declining in Māori populations during the implementation of the other two policies. This revealed the challenge of reducing both nicotine addictions and the highly valued bedsharing custom within one policy, and the need for a new approach [20]. Rather than asking Māori to quit smoking and bedsharing, the new policy set out to reduce the risks of such practices. In other words, the policy shifted from an elimination strategy to risk-minimalization. The safer sleeping solution came through an infant sleeping basket with a mattress known as a wahakura, which could be placed on a couple’s bed and allow for bedsharing with a degree of separation. Combined with advice on how to use the product safely, this strategy dramatically improved infant safety. It also had the added benefit of encouraging parent-child bonding and breastfeeding. As the product was familiar and comfortable for the Māori populations, it was more readily received by them. This also encouraged midwives to pass on recommendations regarding infant safety to other mothers, and this form of communication was subsequently well received. After success in initial trials, the Māori SIDS Prevention Programme began putting money into creating and distributing more of these baskets, and this distribution became a major part of social policy targeting infant mortality inequities.

A problem for the policy in the early stages was that the baskets were difficult to make in the traditional Māori fashion. It soon became apparent that mass manufacturing and distribution was unsustainable without significant funding. This led to the creation and distribution of the ‘pēpi-pod’, which was essentially the same thing but cheaper and easier to make. This became a popular alternative to the original design, especially following the Christchurch earthquake of 2011 which incited fears of isolated infant safety. This prompted the creation of several other policies and variations of baskets, and in this way the benefits of the original wahakura policy were extended among both Māori and non-Māori populations. While the distribution of the wahakuras and pēpi-pods initially went to only a little more than 1000 mothers in the opening months, this distribution created an environment in which their usage became more mainstream and similar devices were readily available to be purchased cheaply across the country, including by non-Māori. This shows that there are two ways to view the expansion of benefits past the initial policy. On the one hand, inequity policy can be thought of as a means to helping the target community through ripple effects which lead to expansion of norms, policy, and better outcomes, as we saw with infant baskets and safety education becoming more popularized. On the other hand, inequity policy can have secondary effects in other sectors of society, as was the case with baby-baskets becoming popular outside of Māori populations.

This third big reform addressing inequities built on the previous reform in several ways, and can be analyzed as follows. Firstly, it was distributed to Māori in a way which did not put any burden on them to acquire the device, and was made easily understandable through simple instructions. Instead of trying to impose the policy on them, the baskets were offered freely and the benefits were clearly articulated. It is unsurprising that easing burdens and simplifying messages enhances the probability of individuals acting as the reform intended. Secondly, the strategic choice of a Māori-made basket increased cultural congruity, building on the earlier 1994 policy’s considerations of Māori inclusion. A willingness to pursue the goals in a different way when the initial efforts fell short was a huge reason this policy was able to successfully target a risk factor which had previously seemed virtually unfixable without massive investment. These two factors led to a high acceptance of the baskets [20], in contrast to the earlier approaches which set out to reduce smoker-infant bedsharing.

This success, alongside the history of infant mortality reform in New Zealand, points to a wider theme in implementing inequity-reduction policy: successful policy tends to follow an evolution of successes and failures. The need for such policy adaption is not unique to welfare issues. For example, Jacob Hacker argues that policy drift can lead to the effects of policy changing over time, which left unaddressed can lead to unintended and undesirable outcomes [21]. Similarly, in his discussion of new social policies, Giuliano Bonoli argues that as societal context changes, policy must adapt to changing societal demands and costs [22]. The New Zealand infant mortality policies extend these observations, in indicating that policy adaptation is not just about addressing changing times and drift, but can also address shortcomings of previous policy.

Most often, the initial form of a policy will not perfectly solve the problems it is targeting, as problems are often multifactorial and unpredictable. The baby-basket reform showed a willingness to improve prior, otherwise successful policies, in the specific areas where those policies were not working. These novel solutions involved cultural sensitivity and risk-alleviation strategies that targeted the underlying risks specific to Māori communities, which demonstrated how compromised policy has the potential to deliver higher compliance and better outcomes. Compared to prior strategies, this approach represented a shift in how successful health policy is implemented.

Overall, this case highlights the importance of reexamining policy as it progresses, and indicates the value of the substantial research effort directed at Māori inequity within New Zealand over recent years. This research emphasis demonstrates New Zealand’s commitment towards long-term reform, which can only be achieved when there is adequate data to consistently assess outcomes. Moving forward, similar adaptive attitudes will prove beneficial in addressing even more difficult infant mortality inequity obstacles such as pregnant smoking and socioeconomic deficiencies.

### Current policy directions

In light of the three major reforms which have taken place over the past several decades concerning infant mortality in New Zealand, there is still an inequity problem, as seen in the higher infant mortality rates of Māori populations (Table 2). However, while additional targeted reform addressing infant mortality does not seem to be on agenda, New Zealand’s government has made recent efforts to improve health inequities across the board.^10^ For example, Labour’s health policy statements from 2014 include “reducing health inequalities” as the first of eight health priorities [24]. Additionally, the latest budgetary conditions are driven by the principle of improving the “well-being” of citizens as opposed to the traditional priorities of productivity or economic growth [25]. This unprecedented move means that new spending must hit at least one of five requirements, one of which is addressing the inequalities faced by indigenous Māori and Pacific islands people [25]. Many of the other requirements are factors which have indirect effects on health disparities, such as reducing child poverty.

These positions and funding shifts are not targeted policy, and they have only been in place a short time, and so it is not yet possible to say if, how, or to what extent they will reduce disparities between Māori and non-Māori populations. It is likely that placing inequity at the forefront of a government’s agenda will promote the benefits of prior reforms, and support further policy and population health initiatives of this kind. This may ultimately lead to further reductions in infant mortality inequities. One may also hope that the broad policy will encourage inclusivity and tolerance, and decrease the negative health effects associated with racism. There are further electoral incentives to tackle these problems, given that the Māori constitute a significant portion of the population of New Zealand [10, 24]. This suggests that the improvements in health equality that have occurred over the past 20 years may continue. Still, as the initial reforms addressing infant mortality demonstrated, policy is unpredictable and it would be premature to assume the exact direction or extent to which these macro shifts will take the problem of health inequity.

### Limitations

There are many valuable lessons to be taken from analyzing the trends, data, and policy of reducing inequities in Māori infant mortality. However, it is also important to acknowledge that other factors may have had a role. Firstly, the New Zealand health system underwent a number of reforms in the same time period, especially during the 1990s. One such reform was the transition to District Health Boards (DHBs), which was a system of funding allocation for different regions of New Zealand. This system included a formula designed to disproportionately favor regions of lower socioeconomic status, which tended to have disproportionately more Māori. It is possible that the higher fund allocations towards Māori-heavy areas could have impacted health inequities in infant mortality, though the favoring of these areas is unlikely to translate into tangible benefits due to the primary causes of infant mortality being SIDS and SUDI, both of which have risk factors associated with the home and outside of the healthcare system. As the policy progression shows, simply putting more money into a problem does not always solve the problem; the money must be used in the proper manner.

Secondly, the definition of “Māori” broadened in 1995, and consequently more people with lower infant mortality were included in subsequent data. This had the effect of making trends immediately following the 1994 policy seem more dramatic than they actually were. For example, the Māori SIDS Prevention Programme saw a reduction of 8 to 3.5 deaths per 1000, though corrected data suggested the actual change being zero. However, this only affects the immediate time period following this specific policy, and the trends continuing past the 1995 transition remain valid.

Finally, there are a multitude of additional factors which affect infant mortality and the broader public’s health. For example, the socioecological framework in public health, the WHO’s Commission on Social Determinants of Health (CSDH) conceptual framework, and the WHO’s Health in All Policies framework all emphasize that role of poverty, poor education, poor housing, and other sociopolitical and contextual elements as inextricably linked to racial health equity [26, 27]. There is also evidence that health inequities are closing between indigenous and nonindigenous populations in other areas such as injuries, which is largely due to changes in physical, social, and economic conditions [28]. Relatedly, many argue that the best way to address infant mortality inequities would be to address social deprivation and poverty, as these are strong predictors of infant health [20]. This would be especially relevant for infant mortality because the risk factors of SIDS and SUDI are bedsharing, smoking, unsafe bedding with back sleeping, and not breastfeeding, all of which are potentially influenced by socioeconomic status. For this reason, social factors changing over time may well have an effect on infant mortality rates just as a targeted policy might. It is impossible to adequately correct for all of these factors, some of which include socioeconomic status, racism, and education. The trend over the past 20 years has been towards more programs targeting Māori inequity in areas which tie back into health outcomes, and it is possible that improvements seen in areas outside of infant mortality policy are reflected in infant health outcomes. That being said, it is highly unlikely that these variations were the reason behind the night and day differences between the initial reform of 1991 and the subsequent reforms of 1994 and on. Because the stagnant trends were so drastically changed following the implementation of 1994 policy, it is still reasonable to conclude that this policy and the inclusive movement it fostered was successful, and that the differences in the second round of policy were important causal factors in addressing inequities.

## Conclusion

An examination of three policies targeting Māori infant mortality inequity provides valuable lessons for future policy which seeks to narrow health gaps between privileged and deprived populations. Specifically, the failure of the initial 1991 reform in addressing Māori infant health, followed by the relative success of post-1994 policy, demonstrated that disadvantaged populations carry differential social risks which require adjusting treatment accordingly. In other words, what works for one community does not always work for another, especially when the other community is socioeconomically disadvantaged, racially discriminated, faces different underlying risk factors, and has a different culture and environment. Policy that sets out to address inequities between populations must draft their policy around differential risk, which requires cultural sensitivity and inclusivity towards disadvantaged groups, as well as a commitment to proportionate universalism in order to appropriately focus efforts to where they are most needed [8]. Additionally, social policy must be willing to adapt to changing circumstances and shortcomings of current policy. For example, the “Back to Sleep” campaign launched in the US in 1994 was highly successful in preventing SIDS, but widened inequities between the those of high and low social class after ignoring differential risks [29]. Similarly, Australia’s 2008 “Closing the Gap” strategy to address infant mortality inequities failed to reduce gaps between Indigenous and non-Indigenous populations, even though infant mortality declined in both groups [30]. As New Zealand, and other countries facing inequities such as the United States and Australia, move forward, they would do well to consider the lessons of New Zealand’s infant mortality policy progressions.

## Data Availability

Not applicable.

## Footnotes

1 Racism towards Māori peoples is now thought to directly hurt the delivery of healthcare and outcomes [3, 4]. For example, there is data to show Māori may be prescribed lifestyle changes over prescriptions at a higher rate than their non-Māori counterparts according to a PHARMAC report published in 2019, with the underlying assumption that Māori are in poor health because of poor choices [5]. Other determinants of health in NZ which have been studied include socioeconomic, environmental/geographical, behavioral, historical, access, representation in health sectors, and quality variation factors [4, 6]. Similar to Australia and other nations with a history of British colonization, New Zealand exhibits patterns of indigenous population disenfranchisement which have carried forward to the present day [6].

2 Amenable mortality is an important measure for inequity among populations, and is defined in the Wai 2575 Māori Health Trends Report as deaths below the age of 75 which could have potentially been avoided with “effective and timely healthcare” [10].

3 According to Stats NZ, Pacific Islander life expectancies were similar to Māori rates. Male life expectancy was 78.7 years for females and 74.5 years for males in 2012-2014 [11].

4 The Health Equity Assessment Tool users guide published in 2008 by the NZ Ministry of Health is an example of a push to cut down on blanket policy which does not necessarily strategically target the underlying problems [12].

5 These programs include the Health and Disabilities Service Act of 1993, the New Zealand Public Health and Disability Act of 2000, and the ever-evolving He Korowai Oranga plan begun in 2002 [13].

6 SIDS, as well as other measures of infant mortality (generic IM and SUDI), all share similar risk factors, so looking purely at SIDS is highly related to the population’s overall infant health.

7 Non-Māori SIDS rates decreased from 3.6 deaths/1000 to 1.6, while Māori counterparts saw decreases of 7.4 to 6.9, as a result of this policy [7].

8 There was evidence to suggest that disproportionately lower rates of Māori taking up smoking, so potentially the campaign would have had a long-term effect here given the easier task of preventing smoking prior to addiction [17].

9 The Māori bedsharing custom was so popular that a study found that 65% of Māori mothers bedshared sometime during the previous night, and 21% of Māori mothers who smoke in pregnancy always or sometimes slept with their infant. Europeans, on the other hand, had 27% bedshare the prior night and 1% of smokers frequently share [18, 19].

10 This is not to discredit the current policies in place designed to tackle infant mortality, as described in this paper, or policy which indirectly affects infant mortality such as the ABC smoking cessation program of 2007 [23].
